# Sodium Hydrosulfide Protects Rats from Hypobaric-Hypoxia-Induced Acute Lung Injury

**DOI:** 10.3390/ijms251910734

**Published:** 2024-10-05

**Authors:** Renjie Wang, Shuhe Ma, Jun Yang, Kai Luo, Qingyuan Qian, Jinchao Pan, Keke Liang, Yihao Wang, Yue Gao, Maoxing Li

**Affiliations:** 1 College of Pharmacy, Gansu University of Chinese Medicine, Lanzhou 730000, China; wangrenjiegy@163.com (R.W.); mclsxka@163.com (S.M.); yangjunzy@163.com (J.Y.); 18294623280@163.com (K.L.); 18394248206@163.com (K.L.); 2 Department of Pharmaceutical Sciences, Beijing Institute of Radiation Medicine, Beijing 100850, China; q15888906749@163.com (Q.Q.); llpp919227@163.com (J.P.); hao_1216@126.com (Y.W.); 3 College of Pharmacy, Lanzhou University, Lanzhou 730000, China; 4 Faculty of Environment and Life, Beijing University of Technology, Beijing 100083, China; 5 National Key Laboratory of Kidney Diseases, Beijing 100850, China

**Keywords:** hydrogen sulfide, high-altitude hypoxia, acute lung injury, proteomics, antioxidant, anti-inflammatory, anti-apoptotic

## Abstract

Hydrogen sulfide (H_2_S), as a key gas signaling molecule, plays an important role in regulating various diseases, with appropriate concentrations providing antioxidative, anti-inflammatory, and anti-apoptotic effects. The specific role of H_2_S in acute hypoxic injury remains to be clarified. This study focuses on the H_2_S donor sodium hydrosulfide (NaHS) and explores its protective effects and mechanisms against acute hypoxic lung injury. First, various mouse hypoxia models were established to evaluate H_2_S’s protection in hypoxia tolerance. Next, a rat model of acute lung injury (ALI) induced by hypoxia at 6500 m above sea level for 72 h was created to assess H_2_S’s protective effects and mechanisms. Evaluation metrics included blood gas analysis, blood routine indicators, lung water content, and lung tissue pathology. Additionally, LC-MS/MS and bioinformatic analyses were combined in performing quantitative proteomics on lung tissues from the normoxic control group, the hypoxia model group, and the hypoxia model group with NaHS treatment to preliminarily explore the protective mechanisms of H_2_S. Further, enzyme-linked immunosorbent assays (ELISA) were used to measure oxidative stress markers and inflammatory factors in rat lung tissues. Lastly, Western blot analysis was performed to detect Nrf2, HO-1, P-NF-κB, NF-κB, HIF-1α, Bcl-2, and Bax proteins in lung tissues. Results showed that H_2_S exhibited significant anti-hypoxic effects in various hypoxia models, effectively modulating blood gas and blood routine indicators in ALI rats, reducing pulmonary edema, improving lung tissue pathology, and alleviating oxidative stress, inflammatory responses, and apoptosis levels.

## 1. Introduction

High-altitude environments are known for their unique characteristics of low pressure and low oxygen, which pose significant threats to human health. In particular, when individuals rapidly ascend to altitudes above 2500 m, their bodies often struggle to adapt to such abrupt environmental changes, leading to a series of physiological responses. To transport oxygen to various organs of the body, the respiratory rate significantly increases, and the heart rate markedly elevates. However, these compensatory mechanisms are insufficient to fully mitigate the effects of low pressure and hypoxia. Moreover, the lung tissue, as the primary organ for oxygen exchange, is subjected to immense stress in the unique environment of low pressure and hypoxia, making it highly susceptible to damage [1,2]. This is manifested by increased pulmonary artery pressure and pulmonary vascular resistance, along with heightened permeability of pulmonary capillary endothelial cells, leading to the extravasation of substantial amounts of fluid into the pulmonary interstitial space, ultimately resulting in edema and acute lung injury (ALI) [3,4]. ALI is a severe pulmonary condition that typically presents with symptoms such as dyspnea, cough, sputum production, and tachypnea, and it can progress to acute respiratory distress syndrome (ARDS) in severe cases. Although significant therapeutic advances have been made in managing ARDS, the mortality rate remains as high as 40% [5,6,7]. Currently, corticosteroids are widely used in drug therapy due to their anti-inflammatory properties. However, this study focuses on acute lung injury (ALI) caused by acute high-altitude hypoxia, which is induced by low-pressure and low-oxygen environments. The mechanisms of this condition include abnormal blood parameters, inflammation, oxidative stress, and exacerbated apoptosis. Therefore, corticosteroids may have limitations in treating high-altitude hypobaric-hypoxia-induced ALI (hereafter referred to as high-altitude hypobaric hypoxia ALI). Since there is no specific drug for high-altitude hypobaric hypoxia ALI at present, this study uses acetazolamide, an effective treatment for altitude sickness, as the positive control group. In summary, the prevention and treatment of high-altitude hypobaric hypoxia ALI has become an urgent issue that requires immediate attention.

The pathogenesis of high-altitude hypobaric hypoxia acute lung injury (ALI) remains unclear; however, oxidative stress and inflammatory responses triggered by hypoxic environments have been identified as key drivers of disease progression [8,9]. Under hypoxic conditions, blood oxygen saturation rapidly declines, prompting a significant increase in red blood cells and hemoglobin to enhance oxygen transport and supply. This process often disrupts the balance between coagulation and anticoagulation systems [10]. Simultaneously, some cells or tissues are forced to intensify their anaerobic metabolism for energy, directly affecting the blood acid–base balance and electrolyte homeostasis, which leads to a range of abnormal markers and clinical symptoms [11]. More critically, reactive oxygen species (ROS) and pro-inflammatory cytokines such as interleukin-1β (IL-1β) and interleukin-6 (IL-6) accumulate rapidly in cells and tissues under hypoxic conditions, potentially impairing the body’s antioxidative and anti-inflammatory systems, thereby exacerbating oxidative stress and inflammatory damage [12]. Therefore, regulating blood parameters, reducing inflammation, and alleviating oxidative stress are crucial for the treatment and prevention of hypobaric hypoxia ALI at high altitudes.

For a long time, hydrogen sulfide (H_2_S) has been considered a significant pollutant in air and water sources, with excessive exposure known to impair the normal function of various organs, including the lungs, brain, and heart [13,14]. However, as research has progressed, there has been a fundamental shift in the understanding of H_2_S’s role. It has been revealed to not only be an endogenous gasotransmitter but also a molecule with various important physiological and pharmacological effects, including the regulation of vasodilation and blood circulation, modulation of cellular energy metabolism, and inhibition of oxidative stress and inflammatory factors [15,16]. Specifically, the literature supports the hypothesis that H_2_S can open K_ATP_ channels on vascular smooth muscle cells (VSMCs), reducing extracellular Ca^2+^ influx [17]; it interacts with nitric oxide (NO) to promote NO release in endothelial cells and works synergistically with NO to relax smooth muscle and to regulate vascular tone [18]. Additionally, H_2_S participates in signaling pathways such as Nrf2, NFκB/iNOS, and HIF-1α to protect against stress-induced lung injury [19] and to mitigate ischemia–reperfusion-induced myocardial injury [20]. Given these findings, we hypothesize that H_2_S may have a beneficial role in the prevention or treatment of hypobaric hypoxia ALI at high altitudes. Unfortunately, the effects and potential mechanisms of H_2_S in hypobaric hypoxia ALI remain unknown. Therefore, this study aims to delve into the role and potential mechanisms of H_2_S in a rat model of acute lung injury under a simulated rapid ascent to high altitude, with the goal of providing new insights and strategies for the prevention and treatment of high-altitude related diseases.

## 2. Results

### 2.1. NaHS Significantly Enhances Hypoxia Tolerance in Kunming Mice

**Figure 1:**
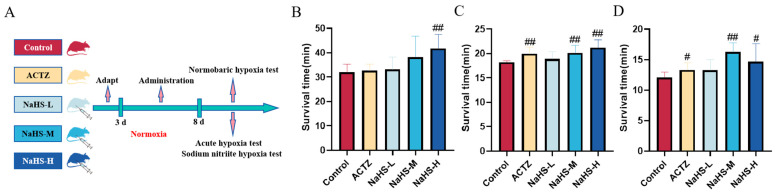
Effects of intraperitoneal NaHS injection on the survival time of hypoxic mice. Data are expressed as the mean ± SD (*n* = 8 per group). Note: ^#^ *p* < 0.05 and ^##^ *p* < 0.01 compared with the Control group. Control: control group; ACTZ: positive-control group; NaHS-L: low-dose NaHS group; NaHS-M: medium-dose NaHS group; NaHS-H: high-dose NaHS group. (**A**) Schematic diagram of the hypoxia model test procedures in mice. (**B**) Normobaric hypoxia test. (**C**) Acute hypoxia test. (**D**) Sodium nitrite-induced hypoxia test.

The experimental procedure is illustrated in Figure 1A. In the normobaric hypoxia experiment, a high dose of NaHS significantly prolonged the average survival time of mice, with the control group averaging 32.0 ± 3.26 min and the NaHS-H group averaging 41.8 ± 5.62 min (*p* < 0.01) (Figure 1B). In the acute hypoxia experiment, both acetazolamide and medium to high doses of NaHS significantly extended the average survival time of mice, with the control group averaging 18.2 ± 0.36 min, the acetazolamide group averaging 19.9 ± 1.07 min, and the NaHS-M and NaHS-H groups averaging 20.1 ± 1.33 min and 21.1 ± 1.00 min, respectively (*p* < 0.01) (Figure 1C). Similarly, in the sodium nitrite hypoxia experiment, acetazolamide and medium to high doses of NaHS effectively extended the average survival time, with the control group averaging 12.1 ± 0.90 min, the acetazolamide group averaging 13.3 ± 0.74 min, and the NaHS-M and NaHS-H groups averaging 16.3 ± 1.62 min and 14.7 ± 3.15 min, respectively (*p* < 0.01 or *p* < 0.05) (Figure 1D). These findings indicate that NaHS significantly enhances the hypoxia tolerance of mice, demonstrating a marked anti-hypoxia effect.

### 2.2. NaHS Protects the Acid–Base Balance and Regulates Ca^2+^ and K^+^ Concentrations in a High-Altitude Hypobaric Hypoxia ALI Rat Model

**Figure 2:**
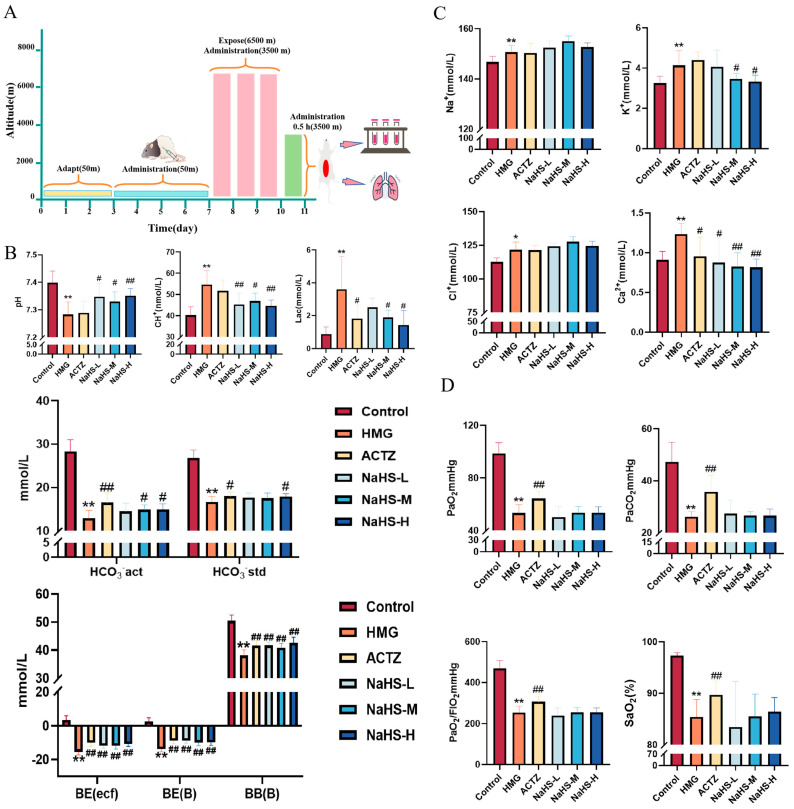
Effects of intraperitoneal NaHS injection on blood gas parameters in high-altitude hypobaric hypoxia ALI rats. Data are expressed as the mean ± SD (*n* = 8 per group). Note: * *p* < 0.05 and ** *p* < 0.01 compared with the Control group; ^#^ *p* < 0.05 and ^##^ *p* < 0.01 compared with the HMG group. (**A**) Schematic diagram of the acute hypoxia rat model experimental procedure. (**B**) Acid–base-related blood gas parameters. (**C**) Electrolyte-related blood gas parameters. (**D**) Oxygen-related blood gas parameters.

The experimental process is illustrated in Figure 2A. The results indicate that, compared with the control group, the model group exhibited significant decreases in acid–base-related parameters, including pH, actual bicarbonate concentration (HCO_3_^−^), standard bicarbonate concentration (HCO_3_^−^), actual base excess (BE(ecf)), standard base excess (BE(B)), and buffer base (BB(B)); in contrast, lactate (Lac) and hydrogen ion concentrations were significantly increased (*p* < 0.05). Electrolyte parameters, such as Na^+^, K^+^, Ca^2+^, and Cl^−^, were significantly elevated in the model group. Oxygen-related parameters, including PaO_2_, PaCO_2_, and the PaO_2_/FIO_2_ ratio, were significantly decreased (*p* < 0.01).

Compared with the model group, all doses of NaHS significantly improved the pH, actual base excess (BE(ecf)), standard base excess (BE(B)), and buffer base (BB(B)) and significantly reduced the hydrogen ion concentration (*p* < 0.05 or *p* < 0.01), while medium and high doses of NaHS significantly decreased lactate levels and increased the actual HCO_3_^−^ concentration (*p* < 0.05) (Figure 2B). Compared with the model group, medium and high doses of NaHS significantly reduced the K+ concentration (*p* < 0.05), and all doses of NaHS significantly decreased the Ca^2+^ concentration, with no significant effect on Na^+^ and Cl^−^ concentrations (Figure 2C). NaHS had no significant effect on oxygen-related parameters, including PaO_2_, PaCO_2_, and the PaO_2_/FIO_2_ ratio (Figure 2D). Overall, NaHS appears to enhance hypoxia tolerance in high-altitude hypobaric hypoxia ALI rats by modulating the acid–base balance and electrolyte levels.

### 2.3. NaHS Modulates Hematological Parameters in High-Altitude Hypobaric Hypoxia ALI Rats

The results indicate that, compared with the control group, the model group exhibited significant increases in white blood cell (WBC), red blood cell (RBC), hemoglobin (HGB), platelet (PLT), and lymphocyte (LYMPH) concentrations (*p* < 0.05 or *p* < 0.01), as well as a significant increase in the hematocrit (HCT) (*p* < 0.05).

**Figure 3:**
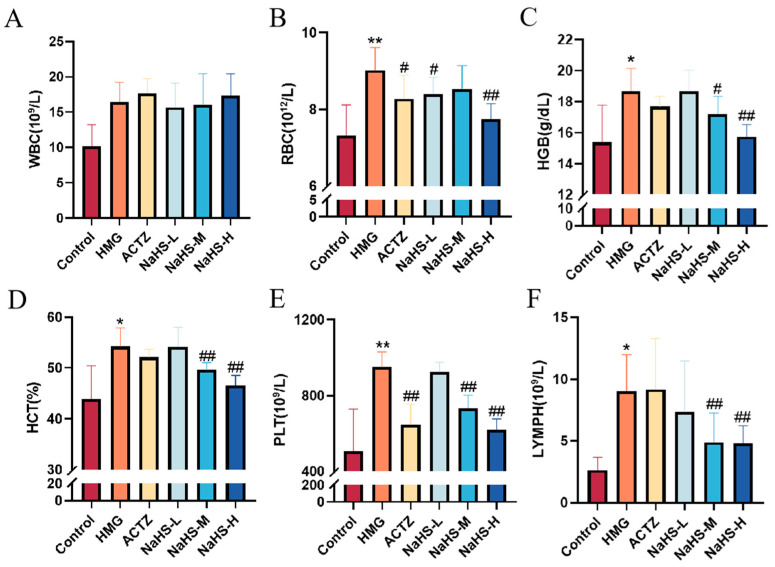
Effects of intraperitoneal NaHS injection on hematological parameters in hypoxia-induced ALI rats. Data are expressed as the mean ± SD (*n* = 8 per group). Note: * *p* < 0.05 and ** *p* < 0.01 compared with the Control group; ^#^ *p* < 0.05 and ^##^ *p* < 0.01 compared with the HMG group. (**A**) White blood cell (WBC) concentration. (**B**) Red blood cell (RBC) concentration. (**C**) Hemoglobin (HGB) concentration. (**D**) Hematocrit (HCT) ratio. (**E**) Platelet (PLT) concentration. (**F**) Lymphocyte (LYMPH) concentration.

Compared with the model group, NaHS treatment significantly reduced the HCT and the concentrations of RBCs, HGB, PLTs, and LYMPHs in the blood of hypoxic rats (Figure 3A–F), with the high-dose NaHS group showing the most pronounced effects (*p* < 0.01). However, NaHS had no significant effect on WBC levels.

### 2.4. NaHS Reduces Lung Water Content and Protects Lung Tissue Structure in ALI Rats Induced by High-Altitude Hypoxia

**Figure 4:**
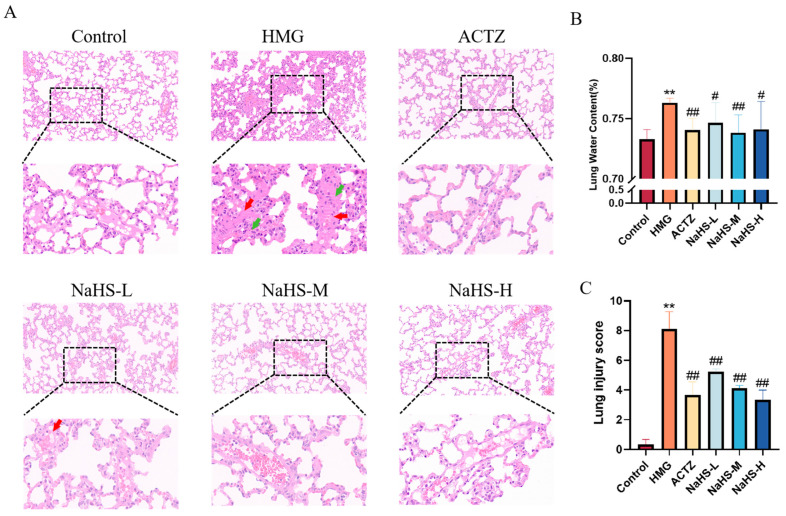
Effects of intraperitoneal NaHS injection on lung tissue structure in rats with ALI induced by high-altitude hypoxia (*n* = 3 per group). Note: ** *p* < 0.01 compared with the Control group; ^#^ *p* < 0.05 and ^##^ *p* < 0.01 compared with the HMG group. Data in panel B are expressed as the mean ± SD (*n* = 6 per group). In panel (**A**), red arrows indicate thrombotic vessels, and green arrows indicate inflammatory cell aggregates. (**A**) Impact of NaHS on lung tissue structure in hypoxic rats. (**B**) Effect of NaHS on lung water content in hypoxic rats. (**C**) Lung injury score.

The results show that, compared with the normoxic control group, the hypoxia model group exhibited an increased number of thrombotic vessels and a higher density of inflammatory cell aggregates in the lung tissue. Both ACTZ and NaHS treatments significantly improved high-altitude hypobaric hypoxia ALI (Figure 4A,C). Additionally, compared with the normoxic control group, the lung water content was significantly elevated in the hypoxia model group, while ACTZ and NaHS treatments notably reduced lung water content (*p* < 0.05 or *p* < 0.01), with the medium dose showing the most pronounced effect (Figure 4B).

### 2.5. Quantitative Proteomic Analysis of Lung Tissue

**Figure 5:**
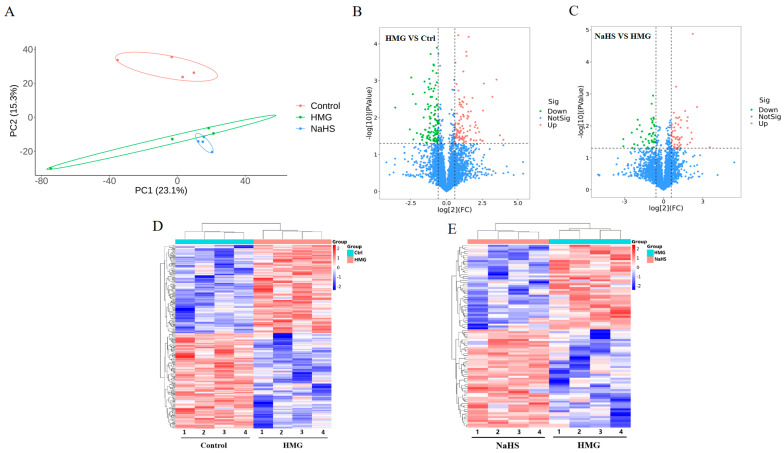
Quantitative proteomic analysis of lung tissue. (**A**) Principal component analysis (PCA) of protein data from the Control, HMG, and NaHS groups. (**B**) Volcano plot of differential proteins between the Control and HMG groups. (**C**) Volcano plot of differential proteins between the HMG and NaHS groups. (**D**) Cluster heatmap of differential proteins between the Control and HMG groups. (**E**) Cluster heatmap of differential proteins between the HMG and NaHS groups.

To explore the protective mechanism of NaHS against high-altitude hypobaric hypoxia ALI, quantitative proteomic analysis was performed on lung tissue samples. Principal component analysis revealed significant differences in protein expression among the Control, HMG, and NaHS groups, indicating that further analysis is warranted (Figure 5A). A total of 216 differential proteins were identified between the HMG and Control groups, with 105 proteins upregulated and 112 proteins downregulated (Figure 5B). Between the NaHS and HMG groups, 95 differential proteins were identified, with 51 proteins upregulated and 45 proteins downregulated (Figure 5C). Cluster heatmap analysis of the differential proteins between the HMG and Control groups, as well as between the NaHS and HMG groups, showed good reproducibility within each group and considerable differences in protein expression among the three groups. Notably, NaHS treatment effectively corrected the protein expression abnormalities observed in the hypoxia model group (Figure 5D,E).

### 2.6. Bioinformatic Analysis of Quantitative Proteomics in Lung Tissue

**Figure 6:**
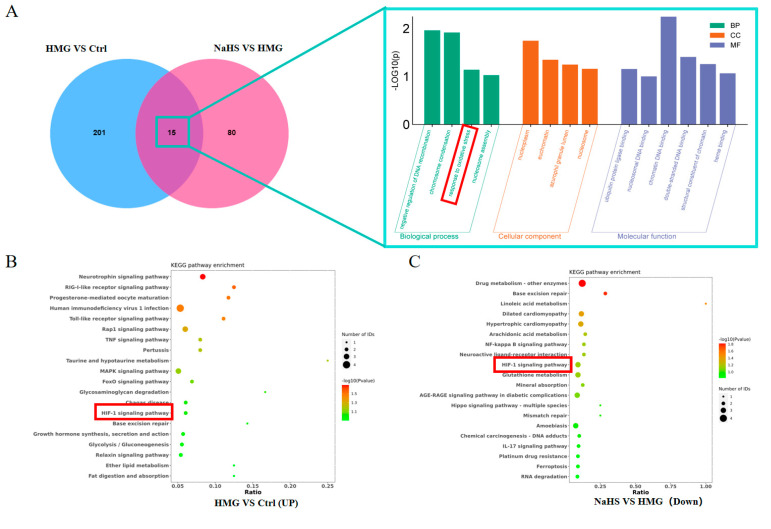

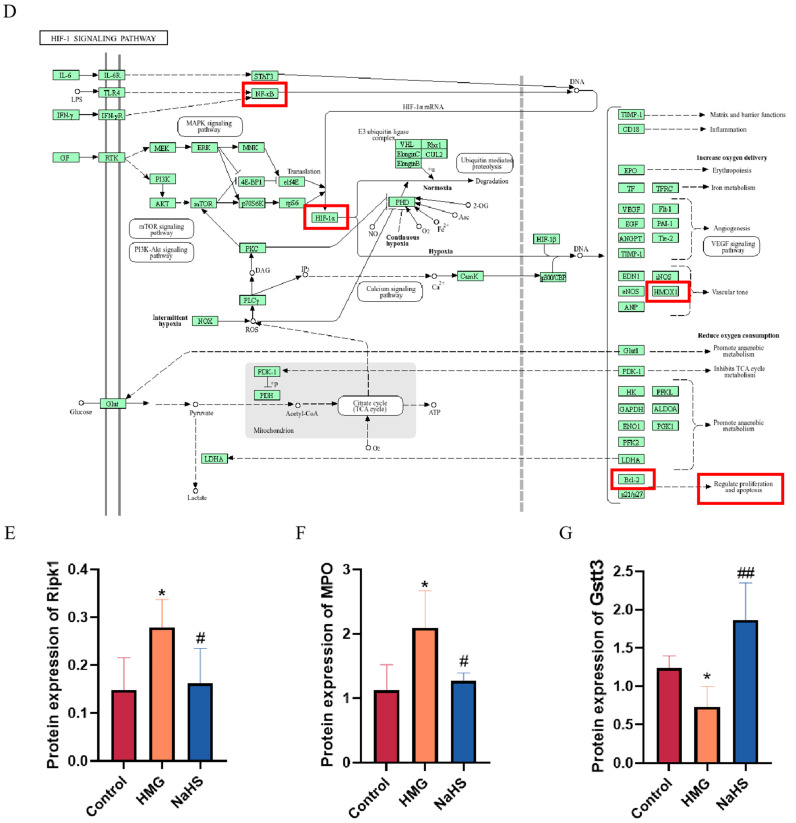
Bioinformatic analysis of quantitative proteomics in lung tissue. Note: Data in panels (**E**,**F**) are presented as the mean ± SD (*n* = 4 per group). * *p* < 0.05 compared with the Control group; ^#^ *p* < 0.05 and ^##^ *p* < 0.01 compared with the HMG group. (**A**) Venn diagram of differential proteins between the HMG and NaHS groups and between the Control and HMG groups, along with GO enrichment analysis of the intersecting differential proteins. (**B**) KEGG pathway analysis of upregulated differential proteins in the Control vs. HMG comparison. (**C**) KEGG pathway analysis of downregulated differential proteins in the HMG vs. NaHS comparison. (**D**) HIF-1 pathway diagram. (**E**) Expression levels of Ripk1 in the quantitative proteomic data across groups. (**F**) Expression levels of MPO in the quantitative proteomic data across groups. (**G**) Expression levels of Gstt3 in the quantitative proteomic data across groups.

Venn analysis of the differential proteins between the HMG and NaHS groups and between the Control and HMG groups identified 15 common proteins (Figure 6A). Gene Ontology (GO) enrichment analysis of these common proteins suggested that NaHS may ameliorate ALI-induced lung damage through oxidative-stress-related pathways.

Further, KEGG pathway analysis of the upregulated proteins in the HMG vs. NaHS comparison and the downregulated proteins in the Control vs. HMG comparison revealed the involvement of the HIF-1 pathway in both analyses (Figure 6B,C). This suggests that NaHS’s protective effect on ALI-induced lung damage may also be associated with the HIF-1 pathway. The mechanistic diagram of the HIF-1 pathway (Figure 6D) indicates that HIF-1 is related to NF-κB and can regulate the HO-1 protein, influencing cellular apoptosis, among other processes. Based on the existing literature on NaHS, we hypothesize that NaHS might improve ALI-induced lung damage through the modulation of oxidative stress, inflammation, and the HIF-1 and apoptosis pathways.

Additionally, analysis of the quantitative proteomics data showed that NaHS improves the expression of proteins associated with oxidative stress and inflammation, such as Ripk1, MPO, and Gstt3 (Figure 6E–G).

### 2.7. NaHS Improves Oxidative Stress and Inflammatory Marker Levels in the Lung Tissue of ALI Rats

**Figure 7:**
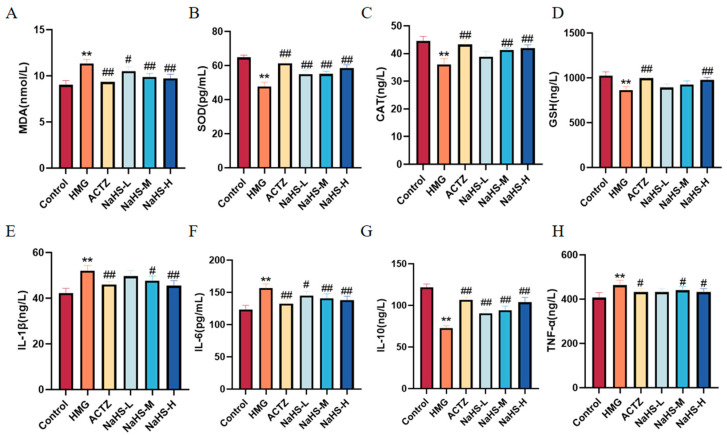
Effects of intraperitoneal NaHS injection on oxidative stress and inflammatory markers in hypoxia-induced ALI rats. Data are expressed as the mean ± SD (*n* = 6 per group). Note: ** *p* < 0.01 compared with the Control group; ^#^ *p* < 0.05 and ^##^ *p* < 0.01 compared with the HMG group. (**A**) MDA levels. (**B**) SOD activity. (**C**) CAT activity. (**D**) GSH levels. (**E**) IL-1β levels. (**F**) IL-6 levels. (**G**) IL-10 levels. (**H**) TNF-α levels.

Oxidative stress and inflammatory markers in lung tissue were assessed. The results indicate that, compared with the Control group, the model group exhibited significantly increased levels of MDA and decreased activities of SOD, CAT, and GSH. Additionally, levels of the inflammatory cytokines IL-1β, IL-6, and TNF-α were markedly elevated, while that of the anti-inflammatory cytokine IL-10 was significantly reduced (Figure 7). Compared with the model group, NaHS at all doses significantly enhanced the activities of SOD, CAT, and GSH, reduced MDA levels, and increased IL-10 levels, while decreasing IL-1β, IL-6, and TNF-α levels (Figure 7). These findings suggest that NaHS mitigates lung damage in ALI rats by modulating oxidative stress and inflammatory markers.

### 2.8. NaHS Protects Rats from Hypoxia-Induced Acute Lung Injury by Regulating Oxidation, Inflammation, and Apoptosis-Related Proteins

**Figure 8:**
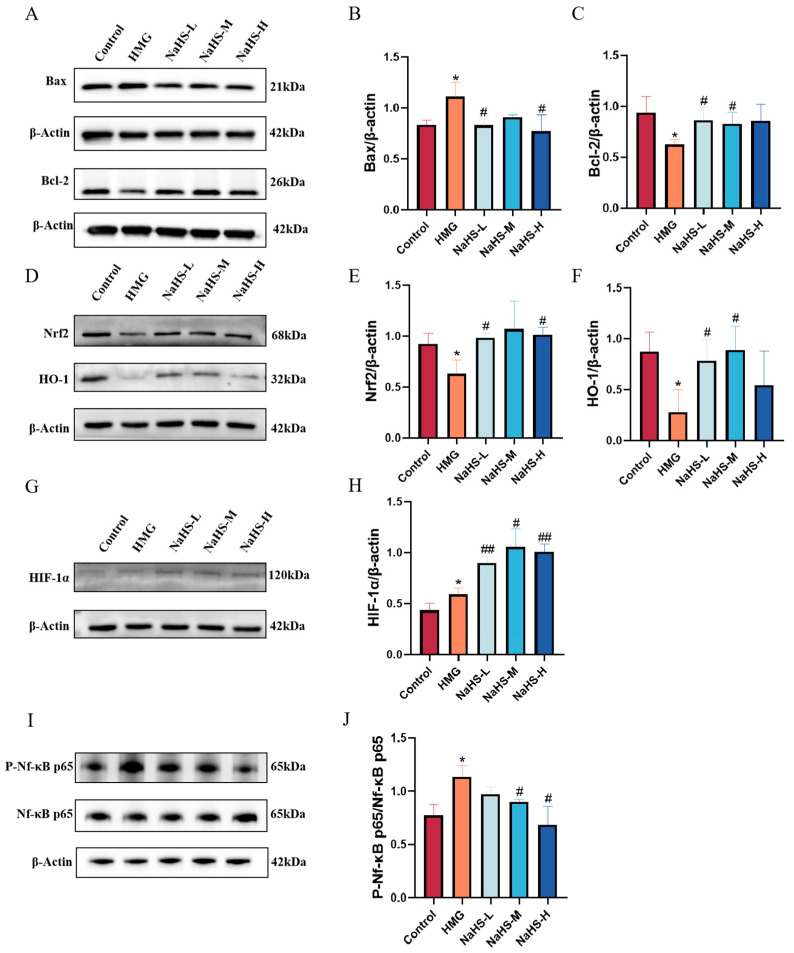
Effect of intraperitoneal NaHS injection on the Nrf2/NF-κB/HIF-1/Bcl-2 signaling pathways in the lung tissue of hypoxic rats. Data are presented as mean ± SD (*n* = 3/group). Notes: * *p* < 0.05 compared with the Control group; ^#^ *p* < 0.05 and ^##^ *p* < 0.01 compared with the HMG group. (**A**) Western blot bands for Bax, Bcl-2, and β-actin. (**B**,**C**) Western blot data for Bcl-2 and Bax were quantified using density analysis. (**D**) Western blot bands for Nrf2, HO-1, and β-actin. (**E**,**F**) Western blot data for Nrf2 and HO-1 were quantified using density analysis. (**G**) Western blot bands for HIF-1α and β-actin. (**H**) Western blot data for HIF-1α were quantified using density analysis. (**I**) Western blot bands for P-Nf-κB p65, Nf-κB p65, and β-actin. (**J**) Western blot data for P-Nf-κB p65/Nf-κB p65 were quantified using density analysis.

Quantitative proteomics indicated that NaHS primarily exerts its protective effects against ALI through antioxidative and anti-inflammatory mechanisms, as well as by modulation of the HIF-1 pathway and apoptosis pathways. Western blot analysis of relevant proteins revealed that, compared with the normoxic control group, Bax expression was significantly increased(Figure 8A,B), Bcl-2 levels were significantly decreased(Figure 8A,C), Nrf2 and HO-1 levels were significantly reduced(Figure 8D–F),HIF-1α and P-Nf-κB p65 expression was significantly elevated(Figure 8G–J). Compared with the model group, NaHS treatment led to significant improvements in these markers (*p* < 0.05 or *p* < 0.01), with the intermediate dose of NaHS showing the most pronounced effect.

## 3. Discussion

The most prominent feature of high-altitude environments is the lower partial pressure of oxygen and thinner air. Upon rapid ascent to high altitudes, the respiratory system is primarily affected, resulting in deeper and faster breathing, an increased heart rate, and enhanced cardiopulmonary interaction, which lead to elevated pulmonary artery pressure. If the body cannot adapt to these compensatory changes due to hypoxia in a short period, it may result in pulmonary dysfunction and, ultimately, progress to acute lung injury (ALI). ALI has a high incidence and mortality rate [21], making it a globally urgent issue. Hypoxia disrupts the balance between oxidation and antioxidation, leading to oxidative stress, and it also triggers inflammatory responses. Studies indicate that inflammation and oxidative stress are closely associated with ALI [22]. Therefore, investigating the roles of oxidative stress and inflammation in hypobaric hypoxia ALI is of significant importance.

Recent research has identified hydrogen sulfide (H_2_S) as the third gaseous signaling molecule in biological systems, following nitric oxide (NO) and carbon monoxide (CO). Although H_2_S was previously primarily recognized for its toxicity, increasing evidence suggests that low concentrations of H_2_S can significantly influence cardiovascular and pulmonary functions through mechanisms involving oxidative stress and inflammation. Sodium hydrosulfide (NaHS) is a well-known exogenous H_2_S donor with a simple structure that is capable of rapidly releasing H_2_S without generating other by-products. Therefore, our aim was to investigate the role of NaHS in the prevention and treatment of high-altitude hypobaric hypoxia acute lung injury (ALI) and to explore its underlying mechanisms.

This study initially evaluated the anti-hypoxic activity of NaHS using models of normobaric hypoxia, acute hypoxia, and sodium nitrite-induced hypoxia in mice. The results indicated that NaHS significantly prolonged the survival time of mice under these hypoxic conditions, demonstrating notable anti-hypoxic efficacy. Further, in a rat model of high-altitude hypobaric hypoxia acute lung injury (ALI), we examined NaHS’s effects on arterial blood gas and hematological parameters. Arterial blood gas analysis, a critical indicator of hypoxic conditions and the efficacy of anti-hypoxic treatments and drugs, is highly sensitive to low-pressure, low-oxygen environments and serves as a key assessment tool for pulmonary function [23,24]. Hematological parameters can also reflect the pulmonary health status, with changes in red blood cell, white blood cell, and hemoglobin concentrations indicating compromised gas exchange efficiency and metabolic disturbances due to pulmonary damage or disease. In high-altitude environments, decreased oxygen partial pressure accelerates respiration and metabolism, leading to reduced arterial oxygen and carbon dioxide partial pressures, electrolyte imbalances, lactic acid accumulation, and elevated red blood cell and hemoglobin levels [25,26]. Therefore, this study used arterial blood gas measurements of PaO_2_, PaCO_2_, and PaO_2_/FIO_2_ to assess the gas exchange function; pH, Lac, H⁺, and HCO_3_^−^ for the acid–base balance; and Na⁺, K⁺, Cl⁺, and Ca^2^⁺ for electrolyte disturbances. Hematological parameters were used to assess pulmonary infection and oxygenation system damage. The experimental results suggest that NaHS may alleviate and mitigate ALI induced by high-altitude hypoxia by modulating the arterial blood gas acid–base balance and electrolyte levels and by regulating oxygen-carrying and immune cells.

Pulmonary water content constitutes 70–80% of the total lung volume. Maintaining the balance of pulmonary water content is crucial for normal lung function, as deviations can impact the gas-exchange efficiency [27,28]. Increased pulmonary water content can result from heart failure, lung inflammation, or acute respiratory distress syndrome. When transitioning from lowland to high-altitude environments, hypoxia induces pulmonary vasodilation, increased vascular permeability, alveolar inflammation, and thrombus formation, ultimately leading to increased pulmonary water content and high-altitude pulmonary edema [29,30]. This study investigated pulmonary water content and pathological changes, revealing that NaHS significantly reduces the pulmonary water content, inhibits thrombus formation, and suppresses inflammatory cell aggregates, thus offering substantial protection against high-altitude hypobaric hypoxia ALI.

**Figure 9:**
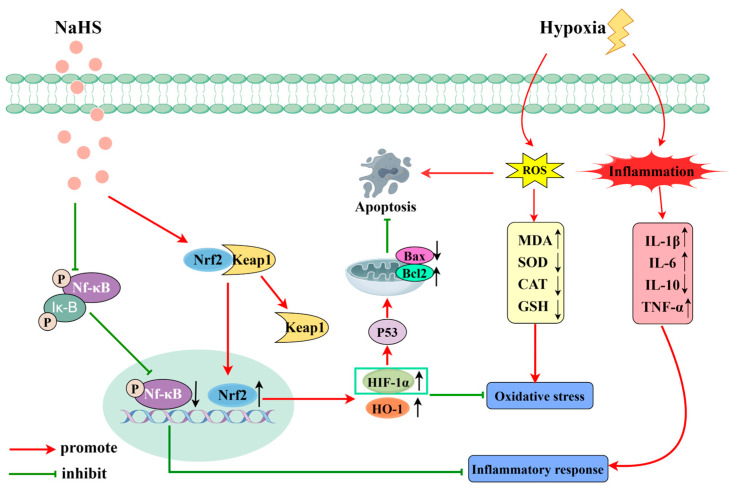
Mechanism of action of NaHS in high-altitude hypobaric hypoxia ALI.

Proteomics involves the comprehensive study of protein composition and activity within cells, tissues, or entire organisms [31,32]. It is significant in biology and medicine, aiding in the discovery of potential biomarkers, early disease diagnosis, and the prediction of disease progression. Additionally, proteomics is crucial for identifying drug targets and for evaluating drug efficacy [33]. This study used proteomics to explore the mechanisms by which NaHS influences high-altitude hypobaric hypoxia ALI. The results indicated that NaHS modulates oxidative stress and inflammatory factors (e.g., significantly reducing Ripk1, MPO, and Gstt3 protein levels in lung tissue) and impacts the HIF-1 signaling pathway, which is involved in cell apoptosis. The activation of Ripk1 protein can further exacerbate oxidative stress, affecting cellular metabolism and mitochondrial function. Additionally, Ripk1 interacts with various inflammation-related pathways, such as the NF-κB and MAPK signaling pathways [34]. The activation of MPO protein can lead to a reduction in antioxidative substances in the body, such as glutathione, resulting in increased oxidative stress and triggering inflammatory responses [35]. The Gstt3 protein is involved in regulating the redox balance, and its elevated levels can promote glutathione synthesis, enhancing the body’s antioxidative capacity [36]. The HIF-1 signaling pathway plays a crucial role in hypoxic environments, helping the body adapt to low-oxygen conditions while regulating the expression of proteins like HO-1 to increase the body’s antioxidative capabilities. It can also modulate the expression of the Bcl-2 protein, reducing the level of cell apoptosis [37,38]. Therefore, we assessed oxidative stress and inflammatory markers in lung tissue. Western blot analysis was performed to measure the levels of the Nrf2, HO-1, NF-κB P65, P-NF-κB P65, HIF-1α, Bax, and Bcl-2 proteins. The results indicated that NaHS exerts antioxidative effects by upregulating Nrf2 and HO-1 protein levels in lung tissue. The ELISA data demonstrated increased activities of SOD, CAT, and GSH and decreased MDA levels in the lungs of ALI rats, further supporting NaHS’s antioxidative properties. NaHS also reduced inflammation by downregulating P-NF-κB P65 levels, as evidenced by lower levels of the pro-inflammatory cytokines IL-1β, IL-6, and TNF-α and increased IL-10 levels. Additionally, NaHS inhibited apoptosis by elevating HIF-1α levels, which upregulated Bcl-2 and downregulated Bax. Overall, NaHS alleviates the acute lung injury induced by hypoxia in rats, likely through mechanisms involving improved oxidative stress, reduced inflammation, and apoptosis inhibition (Figure 9; painting via Figdraw).

In a high-altitude hypoxic environment, the differences in physiological characteristics, hormone levels, and metabolism between males and females can affect experimental results. Therefore, this study used only male mice and male rats as research subjects to minimize variability. However, it is important to note that this cannot fully represent the response of an entire organism to high-altitude hypoxia. In future research, it would be beneficial to include female subjects to gain a more comprehensive understanding of the effects of high-altitude hypoxia on different genders.

## 4. Materials and Methods

### 4.1. Animals, Reagents, and Instruments

SPF male KM mice (4–5 weeks old; average weight 20 ± 2 g) and male SD rats (6–8 weeks old; average weight 200 ± 20 g) were purchased from Beijing Vital River Laboratory Animal Technology Co., Ltd. (Beijing, China, License No. SCXK (Jing) 2021-0011). Animals were housed in a controlled environment with a temperature of 20–25 °C and relative humidity of 55 ± 5%. The light cycle was set to 12 h of light and 12 h of darkness, with ad libitum access to food and water. The experimental protocols used in this study were approved by the Animal Experiment Laboratory of the Military Medicine Research Institute (Beijing, China), with the approval number IACUC-DWZX-2023-P595.

The following lists the reagents used and their source: NaHS (M026633-25 g; Beijing Mireda Technology Co., Ltd., Beijing, China). Pentobarbital sodium salt (product number P3761; Sigma-Aldrich (Shanghai) Trading Co. Ltd, Shanghai, China). Sodium heparin (product number 20220527; China National Pharmaceutical Group Chemical Reagent Co., Ltd., Beijing, China). Acetazolamide (product number RH102875; Shanghai Yien Chemical Technology Co., Ltd., Shanghai, China). DGDR-10-II-type dry electrochemical blood gas and biochemical test cards, along with blood gas testing kits (product numbers W36417401H and W45117127H; Guangzhou Wondfo Biotech Co., Ltd., Guangzhou, China). Urea (product number M123-1KG; Shanghai Jinpan Biotechnology Co., Ltd., Shanghai, China). Protein quantification dye (product number HXJ5137; Beijing Huaxing Bochuang Gene Technology Co., Ltd., Beijing, China). Bovine serum albumin (product number 23209; Thermo Fisher Scientific, Waltham, MA, USA). Dithiothreitol (DTT) (product number M109-5G; Shanghai Jinpan Biotechnology Co., Ltd., Shanghai, China). Acetonitrile (product number 34,851 MSDS; J.T. Baker, Phillipsburg, NJ, USA). Ammonia (product number 013-23355; Wako Pure Chemical Industries Ltd., Chuo-ku, Japan). Formic acid (product number T7970; Sigma-Aldrich, St. Louis, MO, USA). ELISA kits, SOD (product number MM-0386R1), MDA (product number MM-0385R1), CAT (product number MM-20447R1), GSH (product number MM-0602R1), IL-1β (product number MM-0047R1), IL-6 (product number MM-0190R1), TNF-α (product number MM-0180R1), and IL-10 (product number MM-0195R1) were obtained from Jiangsu Meimian Industrial Co., Ltd., Yancheng, China. RIPA lysis buffer (product number P0013C; Biyuntian Biotechnology Co., Ltd., Shanghai, China). BCA protein assay kit (product number CW0014S; Cwbio, Taizhou, China). Antibodies against β-Actin (product number R23613; 1:5000), HIF-1α (product number 340,462; 1:500), Bax (product number 380709; 1:500), and Bcl-2 (product number 381702; 1:500) (Chengdu Zhengneng Bio Co., Ltd., Chengdu, China). Antibodies against Nrf2 (product number 80593-1-RR; 1:2000) and HMOX1 (product number 0701-1-AP; 1:2000) (Wuhan Sanying Biotechnology Co., Ltd., Wuhan, China). Antibodies against NF-κB (product number ab16502; 1:2000) (Abcam, Cambridge, UK). Antibodies against P-NF-κB (product number 3033; 1:1000) (Cell Signaling Technology, Danvers, MA, USA).

DYC-9070 simulated high-altitude, low-pressure hypoxia animal experiment chamber (Fenglei Aerospace & Ordnance Co., Ltd., Anshun, China). BGA-102 portable blood gas and biochemical analyzer (Guangzhou Wondfo Biotech Co., Ltd., Guangzhou, China). Hematology analyzer (Sysmex Medical Electronics Shanghai Co., Ltd., Shanghai, China). ME-204 analytical balance (Mettler-Toledo Instruments Shanghai Co., Ltd., Shanghai, China). E200 optical microscope (Nikon, Tokyo, Japan). RIGOL L-3000 high-performance liquid chromatography system (Beijing Purui Precision Instruments Co., Ltd., Beijing, China). Vortex mixer (SCILOGEX, Rocky Hill, CT, USA). Vacuum concentrator (Model: CV100-DNA; Beijing Jiamu Technology Co., Ltd., Beijing, China). Electric heating constant-temperature water bath (XMTD-7000; Beijing Guangming Medical Instruments Co., Ltd., Beijing, China). Centrifuge (Eppendorf, Germany). ELISA reader (DR200B; Wuxi Huawede Long Instrument Co., Ltd., Wuxi, China). Electrophoresis system (Bio-Rad, Hercules, CA, USA). High-throughput tissue grinder (hf-48; Shanghai He Fan Instrument Co., Ltd., Shanghai, China). Ultrasonic disruptor (JY96-IIN, Shanghai Huxi Industrial Co., Ltd., Shanghai, China).

### 4.2. Normobaric Hypoxia, Acute Hypoxia, and Sodium Nitrite Hypoxia Tests

After acclimatizing 40 Kunming mice weighing 18–20 g for 3 days, these were randomly divided into the following groups: control group (Control), positive drug group (Acetazolamide, ACTZ; 104 mg/kg/day orally), and NaHS low-, medium-, and high-dose groups (NaHS; 4, 8, 16 mg/kg/day intraperitoneally). The control group received an intraperitoneal injection of an equal volume of physiological saline, with a dosage volume of 10 mL/kg. Mice were housed in an environment with a temperature of 23 °C, relative humidity of 60%, and a 12 h light/dark cycle and were provided ad libitum access to sterile food and water. After continuous administration for 5 days in normoxic conditions, experiments were conducted. The following three tests all utilize the same dosing method and grouping.

In the normobaric hypoxia test, 30 min after the last dose, mice were placed in a glass bottle containing 250 mL of sodium lime (5 g) for CO_2_ and moisture absorption. The bottle was sealed immediately, and the survival time of the mice in each group was recorded.

In the acute hypoxia test, 30 min after the last dose, mice were placed in a hypobaric hypoxia simulation chamber, which simulated an altitude increase from 0 m to 10,000 m (with a rate of ascent of 10 m/s). Timing commenced at the start of the ascent, and the survival time of the mice in each group was recorded.

In the sodium nitrite hypoxia tests, 30 min after the last dose, mice in each group were intraperitoneally injected with sodium nitrite (300 mg/kg). Timing commenced immediately after injection, and the survival time of the mice in each group was recorded.

### 4.3. Acute Hypoxic Lung Injury Rat Model

Forty-eight SD rats weighing between 200 and 220 g were acclimatized for 3 days and then randomly divided into the following groups: normoxic control group (Control), acute high-altitude hypoxia model group (HMG), positive drug control group (Acetazolamide, ACTZ; 72 mg/kg/day by oral gavage), and NaHS low-, medium-, and high-dose intervention groups (NaHS; 2.8, 5.6, 11.2 mg/kg/day intraperitoneally). Each group was housed under normoxic conditions for 4 days, during which preventive treatments were administered according to group assignment (normoxic and model groups received the same volume of saline). On the morning of day 5, the model group and all treatment groups were placed in a large hypobaric hypoxia chamber, where they were exposed to a simulated altitude of 6500 m for 3 days with continued drug administration (experimental personnel entered a buffer chamber, which was elevated to 3500 m at a rate of 5 m/s; drug administration was performed when the animal chamber’s altitude reached 3500 m). On day 8, 30 min after the final dose, rats were anesthetized with sodium pentobarbital at a dose of 30 mg/kg. Blood samples were collected from the abdominal aorta of the rats at 3500 m, and lung tissues were harvested. The experimental samples were transported through the pass-through window and the relevant measurements of indicators were performed outside the chamber.

### 4.4. Analysis of Blood Gas Indicators

Animals were immobilized on a surgical table, and an abdominal incision was made. Blood (0.8 mL) was collected from the abdominal aorta using a 1 mL heparinized syringe. The sample was immediately sealed with rubber and analyzed within 5 min using a DGDR-10II dry chemistry blood gas and biochemical testing card on a BGA-102 portable blood gas and biochemical analyzer. The following parameters were measured: pH, PaCO_2_, lactate, PaO_2_, hydrogen ion concentration, actual bicarbonate, standard bicarbonate, standard base excess, actual base excess, buffer base, and PaO_2_/FIO_2_ ratio.

### 4.5. Analysis of Routine Blood Indicators

Blood (1 mL) was collected from the abdominal aorta of each group into heparinized anticoagulant tubes and gently inverted to ensure adequate anticoagulation. Indicators such as white blood cells (WBCs), red blood cells (RBCs), hemoglobin (HGB), and platelets (HCT) were measured using a hematology analyzer.

### 4.6. Analysis of Lung Water Content

The right lung was excised, and the upper lobe was separated. The wet weight of the lung was recorded, with at least six samples per group. Subsequently, the lungs were dried in an oven at 60 °C until a constant weight was achieved, and the dry weight was recorded. Lung water content was calculated using the following formula:

$$Water\;content(\%)=\frac{wet\;weight-dry\;weight}{wet\;weight}\times 100\%$$

### 4.7. Hematoxylin–Eosin Staining of Lung Tissue

The left lung was excised and subjected to hematoxylin–eosin staining to evaluate the pathological morphology of lung tissue. At least three samples per group were used. After fixation, tissues were sectioned, embedded in paraffin, and stained with hematoxylin–eosin. The pathological morphology of each group was observed under a microscope, and pathology sections were scored. Morphological changes were scored as nil (score of 0), mild (score of 1), moderate (score of 2), or severe (score of 3) injury based on five pathological features: (i) the presence of exudates, (ii) hyperemia/congestion, (iii) neutrophil infiltration, (iv) intra-alveolar hemorrhage/debris, and (v) the blood clot level [39]. We invited three pathology experts to provide a comprehensive evaluation of the morphology in a double-blind experiment. Finally, the sum of scores for each rat in each group was averaged.

### 4.8. Preparation of Lung Tissue Protein Samples for Quantitative Proteomic Analysis

A volume of 300 μL of 8 M urea was added to the sample, and protease inhibitor was added at 10% of the lysate. After centrifuging at 14,100× *g* for 20 min, the supernatant was collected. The protein concentration was determined using the Bradford method. A 100 µg aliquot of extracted proteins from each sample was then subjected to reduction by adding 200 mM dithiothreitol (DTT) solution and incubating at 37 °C for 1 h, while the rest of the sample was frozen at -80 °C. The reduced sample was diluted 4 times by adding 25 mM ammonium bicarbonate (ABC) buffer. Then, trypsin (trypsin: protein = 1:50) was added, and the sample was incubated at 37 °C overnight. The next day, 50 μL 0.1%FA was added to terminate the digestion. A volume of 100 μL 100% ACN was used to wash the C18 column, which was then centrifuged at 1200 rpm for 3 min. The column was washed once with 100 μL of 0.1% FA and centrifuged at 1200 rpm for 3 min. The EP tube was replaced, and the sample was added and centrifuged at 1200 rpm for 3 min. The column was washed twice with 100 μL of 0.1% FA and centrifuged at 1200 rpm for 3 min, followed by one wash with 100 μL of pH 10 water. The EP tube was replaced and elution was performed with 70% ACN. The eluents of each sample were combined, lyophilized, and stored at −80 °C until loading.

### 4.9. LC-MS/MS Analysis of Lung Tissue Protein Samples

Nanoflow LC-MS/MS analysis of tryptic peptides was conducted on a quadrupole Orbitrap mass spectrometer coupled to an EASY nLC 1200 ultra-high-pressure system via a nano-electrospray ion source. A total of 500 ng of peptides was loaded onto a 25 cm column (150 μm inner diameter, packed using ReproSil-Pur C18-AQ 1.9- µm silica beads; Beijing Qinglian Biotech Co., Ltd., Beijing, China). The peptides were separated using a gradient from 8 to 12% B over 5 min and then 12% to 30% B over 33 min; this was then stepped up to 40% over 7 min followed by a 15 min wash at 95% B at 600 nl per minute, where solvent A was 0.1% formic acid in water and solvent B was 80% ACN and 0.1% formic acid in water. The total duration of the run was 60 min. The column temperature was kept at 60 °C using an in-house-developed oven. Briefly, the mass spectrometer was operated in the “top-40” data-dependent mode, collecting MS spectra in the Orbitrap mass analyzer (120,000 resolution; 350–1500 *m*/*z* range) with an automatic gain control (AGC) target of 3E6 and a maximum ion injection time of 80 ms. The most intense ions from the full scan were isolated with an isolation width of 1.6 *m*/*z*. Following higher-energy collisional dissociation (HCD) with a normalized collision energy (NCE) of 27, MS/MS spectra were collected in the Orbitrap (15,000 resolution) with an AGC target of 5E4 and a maximum ion injection time of 45 ms. Precursor dynamic exclusion was enabled with a duration of 16 s.

All RAW files were analyzed using the Proteome Discoverer suite (version 2.4, Thermo Fisher Scientific). MS2 spectra were searched against the UniProt Rattus norvegicus proteome database (47,943 target sequences downloaded on 7 March 2023). The Sequest HT search engine was used, and parameters were specified as follows: fully tryptic specificity, a maximum of two missed cleavages, a minimum peptide length of 6, fixed carbamidomethylation of cysteine residues (+57.02146 Da), variable modifications for the oxidation of methionine residues (+15.99492 Da), a precursor mass tolerance of 15 ppm, and a fragment mass tolerance of 0.02 Da for MS2 spectra collected in the Orbitrap. Percolator was used to filter peptide spectral matches and peptides to a false discovery rate (FDR) of less than 1%. After spectral assignment, the peptides were assembled into proteins and further filtered based on the combined probabilities of their constituent peptides to a final FDR of 1%. As the default, the top matching protein or “master protein” is the protein with the largest number of unique peptides and with the smallest value in the percent peptide coverage (that is, the longest protein). Only unique and razor (that is, parsimonious) peptides were considered for quantification.

### 4.10. Bioinformatic Analysis of Differential Proteins

Gene Ontology (GO) and InterPro (IPR) analyses were conducted using the interproscan-5 program against the non-redundant protein database, and KEGG (Kyoto Encyclopedia of Genes and Genomes) was used to analyze the protein family and pathway. The enrichment pipeline was used to perform the enrichment analysis of GO and KEGG, respectively.

### 4.11. Analysis of Oxidative-Stress-Related Markers in Lung Tissue

Oxidative-stress-related markers in lung tissue from each group were quantified using ELISA kits. The analyzed markers included superoxide dismutase (SOD), malondialdehyde (MDA), catalase (CAT), and glutathione (GSH).

### 4.12. Analysis of Inflammatory Cytokines in Lung Tissue

Inflammatory cytokine levels in lung tissue from each group were measured using ELISA kits. The cytokines assessed included interleukin-1β (IL-1β), interleukin-6 (IL-6), interleukin-10 (IL-10), and tumor necrosis factor-α (TNF-α).

### 4.13. Western Blot Analysis

Total protein was extracted from lung tissue using RIPA lysis buffer. Protein concentration was determined with a BCA protein assay kit. Proteins (30 µg per lane) were separated by electrophoresis on a 10% sodium dodecyl sulfate–polyacrylamide gel and transferred to a PVDF membrane. The membrane was blocked with 5% non-fat milk at room temperature for 2 h. It was then incubated with primary antibodies against β-Actin (1:5000), HIF-1α (1:500), Nrf2 (1:2000), HMOX1 (1:2000), NF-κB (1:2000), P-NF-κB (1:1000), Bax (1:500), and Bcl-2 (1:500). Excess primary antibodies were washed away with Tween 20 in Tris-buffered saline (TBS). The membrane was subsequently incubated with appropriate secondary antibodies (1:5000) at 37 °C for 1 h. After washing off excess secondary antibodies with Tween 20 in TBS, protein signals were detected using an enhanced chemiluminescence (ECL) method. Protein band intensities were quantified using ImageJ 1.54i software (National Institutes of Health, Bethesda, MD, USA) and normalized against the internal control protein, β-actin.

### 4.14. Statistical Analysis

GraphPad Prism version 5.0 (GraphPad Software, La Jolla, CA, United States) was used for the statistical analysis. All the data are presented as means ± standard errors of the means. For comparisons between two groups, the Student’s two-tailed *t*-test was employed. Statistical significance was indicated by a *p*-value < 0.05.

## 5. Conclusions

The results of this study indicate that NaHS significantly enhances the hypoxia tolerance in mice and reduces lung tissue damage in hypoxic rats. The underlying mechanisms may be associated with the regulation of oxidative stress, inflammatory responses, and apoptosis. Therefore, an appropriate dose of H_2_S could serve as a therapeutic and preventive agent for altitude-induced acute lung injury (ALI), providing a novel approach for future research on targeted drugs for high-altitude hypoxia. However, there are significant barriers to translating NaHS research into clinical practice. Due to the irritating smell and potential toxicity of H_2_S, directly inhaling or injecting H_2_S as a treatment method poses considerable safety risks, which may harm the respiratory tract and lungs. Additionally, H_2_S is easily oxidized in the air, presenting substantial challenges in the production, storage, and transportation of the drug. Therefore, there is a need to develop suitable dosage forms in the future that can reduce its toxicity and control the release rate and quantity of H_2_S, providing theoretical and feasible strategies for the early clinical application of H_2_S in treating altitude-induced acute lung injury (ALI).

## Data Availability

The data used to support the findings of this study are available from the corresponding author upon request.
